# A pilot study for development of a novel tool for clinical decision making to identify fallers among ophthalmic patients

**DOI:** 10.1186/1472-6947-15-S3-S6

**Published:** 2015-09-04

**Authors:** P Melillo, A Orrico, M Attanasio, S Rossi, L Pecchia, F Chirico, F Testa, F Simonelli

**Affiliations:** 1 SHARE Project, Italian Ministry of Education, Scientific Research and University, Rome, Italy; 2 Multidisciplinary Department of Medical, Surgical and Dental sciences, Second University of Naples, 80138, Italy; 3 School of Engineering, University of Warwick, CV47AL, UK

**Keywords:** Fall risk, fall prediction in elderly, poor vision, Activities of Daily Vision Scale

## Abstract

**Background:**

Falls in the elderly is a major problem. Although falls have a multifactorial etiology, a commonly cited cause of falls in older people is poor vision. This study proposes a method to discriminate fallers and non-fallers among ophthalmic patients, based on data-mining algorithms applied to health and socio-demographic information.

**Methods:**

A group of 150 subjects aged 55 years and older, recruited at the Eye Clinic of the Second University of Naples, underwent a baseline ophthalmic examination and a standardized questionnaire, including lifestyles, general health, social engagement and eyesight problems. A subject who reported at least one fall within one year was considered as faller, otherwise as non-faller. Different tree-based data-mining algorithms (i.e., C4.5, Adaboost and Random Forest) were used to develop automatic classifiers and their performances were evaluated by assessing the receiver-operator characteristics curve estimated with the 10-fold-crossvalidation approach.

**Results:**

The best predictive model, based on Random Forest, enabled to identify fallers with a sensitivity and specificity rate of 72.6% and 77.9%, respectively. The most informative variables were: intraocular pressure, best corrected visual acuity and the answers to the total difficulty score of the Activities of Daily Vision Scale (a questionnaire for the measurement of visual disability).

**Conclusions:**

The current study confirmed that some ophthalmic features (i.e. cataract surgery, lower intraocular pressure values) could be associated with a lower fall risk among visually impaired subjects. Finally, automatic analysis of a combination of visual function parameters (either self-evaluated either by ophthalmological tests) and other health information, by data-mining algorithms, could be a feasible tool for identifying fallers among ophthalmic patients.

## Background

Falls represent a major problem for modern societies given its burden and implication on quality of life and autonomy of elderly and their informal caretakers [1]. The mean and median costs for a fall are about 9,000 and 11,000 euro [2]. Falls are caused by complex and dynamic interactions between intrinsic (subject-based) and extrinsic (environmental) factors [3]. Although over 400 risk factors have been identified [4] and their prioritization remains unclear [5], a commonly cited cause of falls in older people is poor vision. In this regard, several population-based studies have identified poor vision as one of the most frequent risk factors for falls [6-9].

Compared with normal-sighted persons, individuals with visual impairment are almost twice as likely to fall and to have recurrent falls[10]. However, the applicability, sensitivity and particularly, the specificity of subject-specific assessment of falls' risks remain imprecise[11]. For example, several functional mobility tests were proposed in literature to identify subjects at higher risk of falls and their performances were tested and compared showing that none of the test achieved an excellent predictive accuracy for the assessment of falls risk in older people[12]. This could be explained by the fact that the causes of falls are multifactorial with several unrelated to mobility, e.g., poor vision, cardiovascular conditions.

The current paper proposes a novel tool to identify fallers among ophthalmic patients by using data-mining methods applied to vision assessment and questionnaire to achieve information about participants' lifestyle, eye symptoms, use of glasses, systemic medical and ocular surgical history, and current medications.

## Methods

### Study population and ethical approval

This study was conducted on a group of subjects aged 55 years and over, enrolled among the patients visited at the Eye Clinic of the Second University of Naples from February to July 2014. The research followed the tenets of the Declaration of Helsinki and each subject gave informed consent to participate in the study. Ethics approval was obtained from the Institutional Review Board of the Second University of Naples.

Socio-demographic and medical data were recorded with a standardized questionnaire that was developed *ad hoc*, including the variables summarized in Table 1 Table 2 and Table 3. Selected variables, which have been considered in previous studies investigating risk factors for falls, included but were not limited to those on visual impaired subjects [13-23]. In particular, information about lifestyle (e.g., cigarette smoking habit, alcohol consumption, job activity, social engagement, etc), systemic medical history (e.g., history of cancer), and general physical health (e.g. sleep problems, walking aid use, depression, hypertension, diabetes, urinary incontinence, arthritis, Parkinson disease, number and type of prescribed drugs, etc) were recorded. Moreover, a global rating of subjective health was also assessed.

**Table 1:** Socio-demographic information assessed with the structured questionnaire.

*Variables*	*Categories or unit of measures*	*References*
Gender	male; female	[15-17,21]

Age	Years	

Indipendent life	Yes; no	[21]

Health compared with that of age group	Much more healthy; More healthy; About as healthy; Less healthy; Much less healthy	[15]

Living alone	Yes; no	[21]

Type of house	Condominium; single apartment	[21]

Jobs	merchant or craftsman; worker; employed; freelancer; other	[21]

Retired	Yes; no	[13,21]

Frequency pushing/dragging heavy loads	Never; Occasionally; 1-2 per week; Daily; Several times per day	[14]

Attendance at religious service in previous month	Yes; no	[14]

Attendance at club meeting in previous month	Yes; no	[14]

Owns or cares for a pet	Yes; no	[14]

Sufficient contact with family/friends	Sufficient; insufficient	[14]

Ability to raise €350 in an emergency	No difficulty; A little difficulty; Lot of difficulty Impossible to raise €350	[14]

**Table 2:** Medical history information assessed with the structured questionnaire.

*Variables*	*Categories or unit of measures*	*References*
Weight	Kg	[15,16]

Body mass index	Kg / m^2^	[17,18,21]

Smoking habit	yes; no; ex	[15-17,21]

Alcohol consumption	Never; occasionally; usually	[15,21]

Depression	Yes; no	[21,22]

Anxiety	Yes; no	[17]

Urinary incontinency	Yes; no	[21,22]

Osteoarthitis	Yes; no	[16,21,22]

Hypertension	Yes; no	[17]

Diabetes	Yes; no	[16-18]

Hearing loss and/or vestibular problems	Yes; no	[18,21]

Cancer history	Yes; no	[17]

Parkinson disease	Yes; no	[17]

Alzheimer disease	Yes; no	[17]

Asthma	Yes; no	[17]

Cardiovascular disease	Yes; no	[17,18]

Shortage of breath	No; yes; only if going uphill/hurrying	[14]

Problems with headaches	Yes; no	[14]

Problems with Walking	No problem; Uses walking aid; Gait problem (no aid); Nonambulant	[23]

Sleeping hours	Hours	[14]

nocturnal awakenings	Never; often; every night	[14]

waking hour overnight	Hours	[14]

Number of prescribed drugs and types	Antidepressants; antipsychotics; antiemetic; sedatives and hypnotics; medicines for Parkinson's disease; antihypertensive or antiarrhythmic; analgesics; antiepileptic	[15-17,21-23]

**Table 3:** Variables related to eye condition and visual function assessed with the structured questionnaire.

*Variables*	*Categories or unit of measures*	*References*
Ocular conditions	Cataract; pseudophakic; glaucoma; age-related macular degeneration; other retinal degeneration	[20,23]

Use of bifocal / multifocal eyeglasses	Yes; no	[15,23]

Use of eye drops	Yes; no	

Best corrected visual acuity in each eye	Decimals	[17,18,20]

Visual acuity loss	Decimals	[23]

Recent refraction change	Yes; no	[23]

Intraocular pressure	mmHg (average both eyes)	

Better vision	Sunny day; rainy day; indifferent	

Blindness effects	No; entering indoor; exit indor	

Activity of day vision scale: Items: Driving at night Seeing moving objects with night driving Oncoming headlights Daytime driving Drive in unfamiliar areas Read signs at night Read signs during the day See/recognize faces See television Read writing on television Read newspapers Read medicine bottles Read food cans Write checks Thread a needle	5 - no difficulty; 4 - little difficulty; 3- moderate difficulty; 2- extreme difficulty; 1- unable because of poor vision; Not Applicable (considered as missing data)	[24]

All participants attended a baseline assessment where they underwent an eye examination, including assessment of the presence and severity of lens opacities, test of the best-corrected visual acuity (BCVA) by Snellen chart and measurement of the intraocular pressure (IOP). IOP was measured by using Goldmann applanation tonometry and in case of IOP than higher than 20 mmHg the ocular medical records of the patients were reviewed in order to find previous diagnosis of glaucoma, slit-lamp biomicroscopy of anterior segment, fundus examination and computerized visual field were performed to assess if the optic nerve is damaged in order to pose diagnosis of glaucoma. Moreover, if assessed by medical record, recent worsening in the visual acuity or change in the manifested refraction were recorded. Finally, vision problems (e.g. blinding effect when exit from indoor environment, or when entering indoor environment), use of multifocal glasses and of eye drops were asked to each participant.

Visual disability was assessed by the Activity of Daily Vision Scale (ADVS) in the 15-item version proposed by Pesudovs et al.[24]. For each item, the patient was asked whether if he/she engaged in the activity (if not it is "Not Applicable" which is treated as missing data), and then the level of difficulties in doing the activity: no difficulty (5), a little difficulty (4), moderate difficulty (3), extreme difficulty (2), unable to perform the activity because of poor vision (1). Finally, the average score for the 15 items was computed.

A fall was defined as unintentionally coming to the ground or some lower level not as a result of a major intrinsic event (e.g., stroke) or overwhelming hazard; participants were asked to report any fall in the previous year and, consequently, they were classified as fallers or non-fallers for the purposes of the current study. Moreover, after the baseline assessment, participants were contacted by telephone in order to record any falls experienced over a prospective 12-month follow-up.

### Data-mining methods

Three different data-mining approaches were used to develop classifier for faller identification, i.e. the C4.5 decision tree induction algorithm, the Random Forest (RF), and the boosting meta-learning approach AdaboostM1 (AB).

The choice of the algorithm parameters was based on the performances (i.e. accuracy, then sensitivity and finally specificity) estimated by 10-fold cross-validation: one with 90% subjects for training and the other with 10% subjects for validation. Repeating the test 10 times, the classification performance were then calculated by averaging the values obtained from the 10 validation subsets.

C4.5 is the landmark decision tree algorithm developed by Quinlan et al.[25]. The feature of each node is selected in order to divide input samples effectively and information gain is used as a measure of effectiveness. After the induction of the decision tree, a pruning method was applied to reduce the tree's size and complexity.

RF is the state-of-the-art classifier developed by Breiman[26]. It is composed of a number of decision trees that choose their splitting attributes from a random subset of *k *attributes at each internal node. The best split is taken among these randomly chosen attributes and the trees are built without pruning, as opposed to C4.5. One of the most relevant downsides of using RF, particularly in medical domain data-mining, is that its model is not easily understandable as a single tree. Moreover, we computed the feature importance measures based on Random Forests (RF)[26].

AB is a meta-learning algorithm which works by incrementally running classifiers on samples of data instances and combining them into an aggregate model[27]. Each individual or weak classifier contributes to the aggregate model in proportion to its accuracy. After each iteration, data instances are reweighted based on incorrect aggregate classifications. This boosts the emphasis of misclassified instances, refining the construction of weak classifiers in future iterations. In the current study, C4.5 was adopted as weak classifier in the AB algorithm.

AB classifiers were developed by varying the number of iteration from 20 to 400 and C4.5 trees (both as single classifier and as base classifier in AB) were developed by varying confidence factor for pruning from 0.05 to 0.5, minimum number of instances per leaf from 5 to 20. MLP were trained by varying the learning rate from 0.3 to 0.9, the momentum from 0.2 to 1 and the number of epoch form 100 to 2000. RF was constructed using an ensemble of random trees from 20 to 400 with no depth limit and varying the number of randomly chosen features from log_2_(*n*)+1 to *n*, where *n *is the number of feature. As regards SVM, we used radial basis function kernel, varying gamma from 10^-5 ^to 10.

## Results

The study sample consisted of 150 participants (mean age ± standard deviation: 73.0 ± 9.6 years; range: 55-99 years) including 60 males (40%) and 90 females (60%). Participants had a range of severity of visual impairment, for example, BCVA ranging from no light perception to 20/20. 109 participants (72.7%) suffered from cataract in at least one eye, whereas 42 participants (28.0%) were pseudophakic in at least one eye.

**Figure 1:**
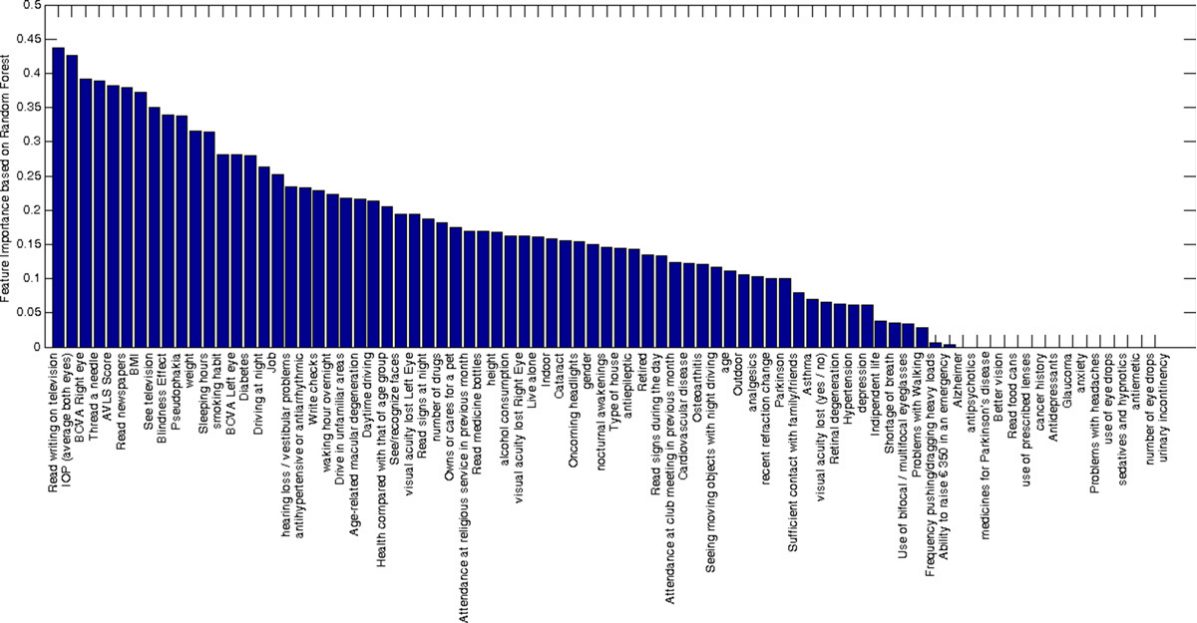
**Feature Importance of variables estimated by Random Forest**. Among the ten most informative variables, five were obtained by the ADVS questionnaire; the others five were BCVA, IOP, BMI, blindness effect and weight. ADVS: Activity of day vision scale; BCVA: Best Corrected Visual Acuity; IOP. IntraOcular Pressure; BMI: Body Mass Index.

The most informative variables, according to the values of feature importance estimated by RF, were: the answer to the item "Read writing on television" of the ADVS, IOP and BCVA in right eye. As shown in Figure 1, among the ten most relevant variables, five were obtained by the ADVS, i.e. the difficulty score, and the following items: "See television"; "Thread a needle"; "Read writing on television"; "Read newspapers".

**Figure 2:**
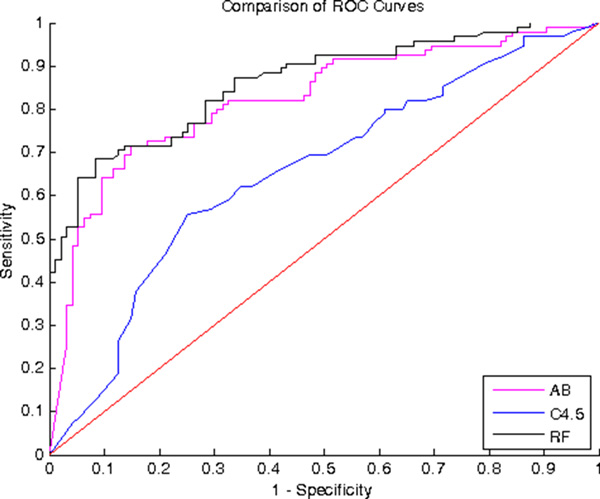
**Comparison of the Receiver Operating Curves of the selected classifiers**. The RF achieved better performances in terms of Area Under the Curve than C4.5 and AB. RF: Random Forest; AB: AdaBoost.

For each data-mining method, the optimal combination of parameters were selected by maximizing the accuracy estimated by 10-fold-crossvalidation as shown in Table 4. The ROC curves for identifying fallers are compared in Figure 2. RF and AB outperformed C4.5 in terms of overall accuracy, sensitivity and specificity rates. RF achieved slightly better performances than AB.

**Table 4:** Classification performances of the selected classifiers.

Classifier	parameters	Accuracy (95% CI)	Sensitivity	Specificity	AUC
AB	NI: 200; CF: 0.5; ML: 25	74.7 % (71.1% - 77.4%)	75.8 %	73.7 %	82.3 %

C4.5	CF: 0.4; ML: 20	63.7 % (60.0% - 66.9%)	57.9 %	69.5 %	65.5 %

RF	NT = 200; NV = 15	75.3 % (71.7% - 77.9%)	72.6 %	77.9 %	86.2 %

CI: Confidence Interval;

AB: Adaboost;

RF: Random Forest;

NI: number of iteration;

ML: minimum number of instances per leaf;

CF: confidence factor for pruning;

NT: number of trees;

NF: number of randomly chosen features.

**Figure 3:**
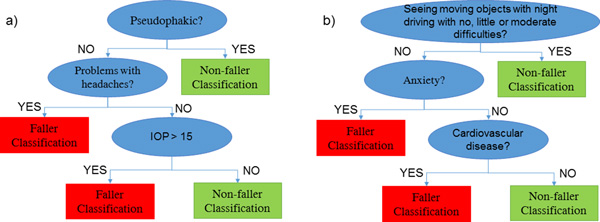
**Examples of classification tree developed in the Adaboost algorithm. a) **The tree indicated that the subject is classified as a faller in case of pseudophakia associated with headaches or IOP higher than 15 mmHg; otherwise the subject is classified as non-faller**. b)**The tree indicated that the subject is classified as a faller in case of extreme difficulties or inability to seeing moving objects with night driving associated with anxiety or cardiovascular disease; otherwise the subject is classified as non-faller.

Since AB achieved the highest sensitivity, it was interesting to observe the rules obtained from the decision tree with the highest weights, including or not including ophthalmic features, shown in Figure 3. According to the decision tree including ophthalmic features, the subject was labelled as non-faller if pseudophakic, otherwise, in case of headache problems or IOP higher than 15, the subject was classified as faller. According to the model without any ophthalmic feature (Figure 3a), if the subject referred no or little or moderate difficulties in "Seeing moving objects with night driving", the non-faller label was assigned; otherwise, the classification was based on the presence of anxiety or cardiovascular disease: in case of anxiety and/or any cardiovascular disease, the subject was identified as fallers, whereas the subjects not suffering from anxiety nor any cardiovascular disease were classified as non-fallers.

## Discussion

This paper presented a pilot study to develop a novel tool to identify fallers among ophthalmic patients, based on a few ophthalmological parameters (such as ocular disease, BCVA, IOP) and a standardized questionnaire (including self-evaluation of visual ability). The system has been realized by comparing different approaches for developing decision tree. Each algorithm achieved a satisfactory performance, e.g. the area under the curve is higher than the performance of random choice (i.e. 0.5) and the Random Forest, the state of art classifier based on decision tree, achieved the best performance, with sensitivity and specificity rate of 72.6% and 77.9%, respectively.

The comparison between ROC curve of the proposed method and the performance of several functional mobility tests for predicting falls in community-dwelling older people showed that the proposed method achieved higher sensitivity and specificity rates than all the functional tests, which had relative risk (RR) ranging from 1.3 to 2.3 and sensitivity and specificity scores ranging from 11% to 78%, and 28% to 93%[12]. Moreover, these tests need that the subject could perform a mobility action, for that reason, they could be not suitable for all subjects with visual impairment and, finally, they requires materials and expertise, which are not usually available in an eye clinic. On the contrary, the proposed methods required only few ophthalmological parameters, such as IOP and BCVA, which are routinely measured in eye clinics, and the assessment of a questionnaire, which could filled in part by the physician and in part by the patient (under physician supervision) in about 30 minutes. The developed questionnaire strongly relies on a standardized questionnaire (ADVS), which have been developed for the evaluation of outcome of cataract surgery. We adopted a reduced ADVS version, since it has been shown to have an adequate precision, equivalent criterion validity, improved targeting of item difficulty to patient ability with decreased time for filling the questionnaire[24].

The current study has some limitations, in particular, the small sample size, Therefore, the clinical implications of these findings are potentially relevant, since the requested parameters are based on simple and non-invasive measurements, even if an external and further validation on a large dataset is required. For that reason, a further development of the current study could be the test of a reduced questionnaire including only the most significant variables submitted to a large study sample. Finally, the tool for identification of fallers could be integrated in the web-based platform, developed in the framework of the Smart Health and Artificial Intelligence for Risk estimation (SHARE) project[28]. The platform, now integrated in an open and interoperable cloud computing platform for health and eGovernement (PRISMA), will enable to test the clinical feasibility and uptake of the developed tool in a prospective study. The system has been already tested for cardiovascular disease severity assessment[29] and cardiovascular risk assessment[30]. Moreover, since the most variable could be collected without any specialist expertise, future research will focus on the clinical applicability of the system as a screening tool in non-specialized ambulatories (e.g. at General Practitioners'), in order to identify high-risk patients to be shortlisted for more complex (and costly) investigations. The method could be enhanced by the adoption of instrumental tests[31]. Improved identification of visually impaired individuals at high fall risk may result in more targeted and adequate prevention strategies.

## Conclusions

This study proved that visual assessment and a standardized questionnaire, including the ADVS self-evaluation of visual impairment, could be useful for the automatic identification of fallers among the ophthalmic patients. The developed model enabled to identify fallers among ophthalmic patients with sensitivity and specificity rates of 71.4% and 87.8%, respectively. These findings pave the way to the development of a novel tool for assessment of fall risk among patient with visual impairment.

## Competing interests

The authors declare that they have no competing interests.

## Authors' contributions

PM analyzed the data and wrote part of the manuscript. AO interpreted the data and wrote part of the manuscript. AO and MA contributed to the study design. LP contributed to data analysis. FC performed the electronic data collection. SR, FT, and FS conceived of the study, supervised the study and contributed to data interpretation and critically revised the manuscript for important intellectual content. All the authors read and approved the final version of this paper.

## Declarations

Publication costs were funded by "the 2007-2013 NOP for Research and Competitiveness for the Convergence Regions (Calabria, Campania, Puglia and Sicilia)" with code PON04a3_00139 - Project Smart Health and Artificial intelligence for Risk Estimation.

This article has been published as part of *BMC Medical Informatics and Decision Making *Volume 15 Supplement 3, 2015: Multidimensional, multidiscipline and shared decision making in healthcare and eHealth. The full contents of the supplement are available online at http://www.biomedcentral.com/bmcmedinformdecismak/supplements/15/S3.
